# Telemedicine with clinical decision support for critical care: a systematic review

**DOI:** 10.1186/s13643-016-0357-7

**Published:** 2016-10-18

**Authors:** Nicola Mackintosh, Marius Terblanche, Ritesh Maharaj, Andreas Xyrichis, Karen Franklin, Jamie Keddie, Emily Larkins, Anna Maslen, James Skinner, Samuel Newman, Joana Hiew De Sousa Magalhaes, Jane Sandall

**Affiliations:** 1 Division of Women’s Health, Faculty of Life Sciences and Medicine, Women’s Health Academic Centre, King’s Health Partners, King’s College London, 10th Floor North Wing, St Thomas’ Hospital, Westminster Bridge Road, London, SE1 7EH UK; 2 Guy’s and St Thomas NHS Foundation Trust, London, UK; 3 Division of Health and Social Care Research, King’s College London, London, UK; 4 King’s College Hospital NHS Foundation Trust, London, UK; 5 Florence Nightingale Faculty of Nursing and Midwifery, King’s College London, London, UK

**Keywords:** Telemedicine, Critical care, Patient safety, Clinical decision support, Systematic review

## Abstract

**Background:**

Telemedicine applications aim to address variance in clinical outcomes and increase access to specialist expertise. Despite widespread implementation, there is little robust evidence about cost-effectiveness, clinical benefits, and impact on quality and safety of critical care telemedicine. The primary objective was to determine the impact of critical care telemedicine (with clinical decision support available 24/7) on intensive care unit (ICU) and hospital mortality and length of stay in adults and children. The secondary objectives included staff and patient experience, costs, protocol adherence, and adverse events.

**Methods:**

Data sources included MEDLINE, EMBASE, CINAHL, Cochrane Library databases, Health Technology Assessment Database, Web of Science, OpenGrey, OpenDOAR, and the HMIC through to December 2015. Randomised controlled trials and quasi-experimental studies were eligible for inclusion. Eligible studies reported on differences between groups using the telemedicine intervention and standard care. Two review authors screened abstracts and assessed potentially eligible studies using Cochrane guidance.

**Results:**

Two controlled before-after studies met the inclusion criteria. Both were assessed as high risk of bias. Meta-analysis was not possible as we were unable to disaggregate data between the two studies. One study used a non-randomised stepped-wedge design in seven ICUs. Hospital mortality was the primary outcome which showed a reduction from 13.6 % (CI, 11.9–15.4 %) to 11.8 % (CI, 10.9–12.8 %) during the intervention period with an adjusted odds ratio (OR) of 0.40 (95 % CI, 0.31–0.52; *p* = .005). The second study used a non-randomised, unblinded, pre-/post-assessment of telemedicine interventions in 56 adult ICUs. Hospital mortality (primary outcome) reduced from 11 to 10 % (adjusted hazard ratio (HR) = 0.84; CI, 0.78–0.89; *p* = <.001).

**Conclusions:**

This review highlights the poor methodological quality of most studies investigating critical care telemedicine. The results of the two included studies showed a reduction in hospital mortality in patients receiving the intervention. Further multi-site randomised controlled trials or quasi-experimental studies with accompanying process evaluations are urgently needed to determine effectiveness, implementation, and associated costs.

**Trial registration:**

PROSPERO CRD42014007406

**Electronic supplementary material:**

The online version of this article (doi:10.1186/s13643-016-0357-7) contains supplementary material, which is available to authorized users.

## Background

Population-based studies in the developed world suggest that the burden of critical illness is higher than appreciated and will increase as the population ages [1]. Existing critical care structures and organisational processes are perceived as inadequate to efficiently support these demands [2, 3].

Access to critical care specialists is also not provided for a number of patients in rural areas, and some hospitals provide only daytime intensivist^1^ cover [4]. Implementation of evidence-based guidelines remain problematic [5, 6], and outcomes for critically ill patients demonstrate variation at the hospital, regional, and national levels [7, 8]. Safety culture varies widely across ICUs [9], and medical errors are common in critical care settings due to the fast-paced, complex nature of the work and the vulnerability of the patients [10, 11].

Telemedicine is proposed as a potential solution to address these structural inadequacies in critical care resource and variability in clinical outcomes and access to specialist expertise across units. Telemedicine is the use of telecommunications technology for medical diagnosis and patient care [12]. It offers a solution to structural problems affecting access to care [13, 14] and an additional safety net to support existing services. Critical care telemedicine uses a remotely located support centre housing a critical care team who are networked with a number of bedside critical care unit teams and patients via audio-visual communication and computer systems over the 24-h period. It offers the potential for multiple opportunities for safety and quality improvement on account of off-site support provided by intensivists and critical care nurses, continuous monitoring with early warning capabilities, rounding tools to monitor at-risk patients, inbuilt clinical decision support, and prompts regarding adherence to best practice.

Two recent Cochrane reviews have concluded there is little robust evidence about telemedicine’s cost-effectiveness, clinical benefits, and impact on quality and safety [12, 15]. These reviews focused on telemedicine applications that involve *direct* patient care, in which the patient is remote from the clinician. The telemedicine delivers clinical information and permits consultation and discussion between healthcare professionals and patients regardless of where the patient is located, for example, the remote monitoring of patients with chronic conditions at home, the provision of specialist consultations for patients via video-conferencing, and the provision of clinical information for patient self-management [12, 15].

Missing from these reviews are studies of telemedicine applications involving provider-provider interaction either in addition to or substituting for usual care. Critical care telemedicine is one such model as it provides remote specialist provider support to bedside staff while also involving the patient at the point of care. Existing reviews focusing on critical care telemedicine have so far been limited methodologically [16–19] and have merged together diverse models of application, thus limiting their ability to reach meaningful conclusions about clinical and service utility. Currell et al. [12] and Flodgren et al. note that in order to answer questions about the efficacy of telemedicine, reviews need to focus on particular study populations and intervention functions, as well as staffing models and healthcare systems involved in delivering the intervention.

The primary objective of this review is to compare the effect of 24-h telemedicine models of critical care with standard models of care for acutely ill adults and children.

## Methods

The review adhered to recommendations in the Preferred Reporting Items for Systematic Reviews and Meta-Analyses (PRISMA) Statement [20] and used Cochrane methods guidance [21]. The review protocol is registered on the PROSPERO prospective register of systematic reviews (CRD42014007406).

### Criteria for considering studies for this review

#### Types of studies

All randomised controlled trials, quasi-randomised controlled trials, controlled before-after studies, and interrupted time series studies which evaluated critical care telemedicine were included in the review [22]. We took guidance from the EPOC resources to determine the criteria for including studies employing these designs [23].

#### Types of participants

The population included any adults or children of either gender or any age, or ethnic group admitted to a critical care setting (this included coronary care, high dependency, and intensive care). All conditions and grades of acuity/severity of illness were included within the study population. Acutely ill adults and children cared for outside critical care settings were excluded.

#### Types of interventions

Studies were considered eligible for inclusion if the telemedicine intervention included (1) continuous electronic recording of patients’ vital signs at the bedside which was linked to a computer system enabling display of real-time data and (2) use of clinical decision-making algorithms and electronic alerts by (3) a remotely located team of critical care specialists including doctors, available 24/7. The review excluded telemedicine applications that were periodic (e.g. intermittent rounding or video consultations) or excluded medical decision-making (e.g. nurse-led remote screening of best practice).

#### Types of outcome measures

##### Primary outcomes

The primary outcomes were ICU and hospital mortality and length of stay.

##### Secondary outcomes

Additional outcomes were adverse events, staff and patient experience, costs, and protocol adherence.

### Search methods for identification of studies

#### Electronic searches

The database search strategy is detailed in Table 1 and was conducted through to December 16, 2015; no date or language restrictions were imposed. Databases searched were MEDLINE, EMBASE, CINAHL (Cumulative Index to Nursing and Allied Health Literature), the Cochrane Library, Health Technology Assessment Database, and Web of Science (science and social science citation index). The search strategy was applied consistently across the databases. To avoid publication bias, we also searched for grey literature using OpenGrey, OpenDOAR (the Directory of Open-Access Repositories), and the HMIC (Healthcare Management Information Consortium).

**Table 1:** Search strategy

1. eICU.mp.	
2. tele-ICU.mp.	
3. tele-intensive care.mp.	
4. telemonitoring.mp	
5. OR/1-5	
6. Exp critical care/or critical care.mp.	
7. Exp intensive care/	
8. Exp intensive care units/or intensive care unit$.mp.	
9. Exp Coronary Care Units/or coronary care unit$.mp	
10. coronary care.mp	
11. high dependency.mp	
12. OR/7-12	
13. 6 and 13	

#### Searching other resources

In addition, we hand searched journals, conference proceedings, and journal supplements for papers related to telemedicine in critical care. Forward citation searching of relevant papers was also utilised.

### Data collection and analysis

#### Study selection

The titles and abstracts of each study were independently reviewed and assessed for eligibility by a team of reviewers (NM with JS/AM/JS/EL). We used Covidence (an online systematic review production platform) to systematise the screening process through independent assessment from two reviewers. Any disagreement or lack of consensus was resolved through consultation with two other authors (MT and RM).

#### Data extraction and management

Data extraction was carried out independently by KF, JK, SN, AM, JS, and EL using a standardised, pre-piloted form. Extracted information included country; study setting including size and type of hospital, university affiliation, and teaching status; critical care setting including type of unit and specialisation, study population, and participant demographics; details of baseline conditions including unit staffing ratios of medical and nursing staff and allied health professionals and utilisation of information technology; details of the intervention including the nature of telemonitoring and clinical decision support, location of the telemedicine support centre, and staffing ratios and roles; commercial sponsorship and study methodology; recruitment and study completion dates; outcomes and times of measurement; and suggested mechanisms of intervention action.

#### Assessment of risk of bias in included studies

Study quality was assessed by the same team using the risk of bias criteria for EPOC reviews [24]. We collected information about allocation, baseline measurements, blinding, reliability of primary outcomes, protection against contamination, and other risks of bias. Any discrepancies identified were resolved through discussion with NM.

#### Data synthesis

We used Review Manager 5 (The Cochrane Collaboration, Oxford, England) for data synthesis. In the results, we report the following data: pre-intervention and telemedicine data and statistical significance across groups, absolute and percentage improvement, and adjusted odds and hazard ratios. Meta-analysis was not possible as one of the studies was also included within the dataset of the second study. Following correspondence with the authors, it was not possible to disaggregate the data from the two studies with sufficient confidence to use meta-analysis to pool results. Instead, we present the results of the studies and make a qualitative assessment of the effects of both studies, based on quality.

## Results

### Results of the search

**Fig. 1:**
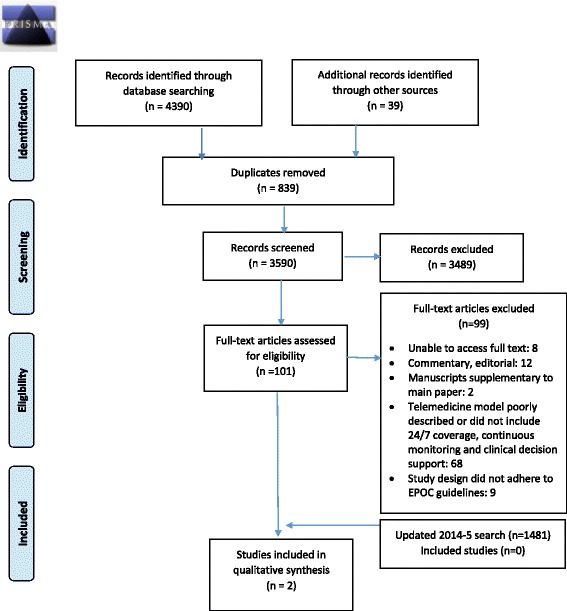
PRISMA flow diagram

The initial database search in February 2014 identified 4390 studies with 39 additional records identified through other sources. An updated 2014–2015 search identified a further 1481 studies (Fig. 1 PRISMA flow diagram and Additional file 1 PRISMA Checklist). One hundred one full-text reports were reviewed. Four authors were contacted for details about the telemedicine model. The studies evaluated a wide variety of interventions, usually with very brief descriptions of the intervention. Once we excluded commentaries and studies that restricted their telemedicine models to remote screening of best practice adherence, intermittent rounding models, or limited intensivist coverage to night-time coverage, we were left with 11 potential studies that included continuous critical care telemedicine models with 24-h coverage.

### Excluded studies

Nine studies were excluded as the study designs did not meet the EPOC criteria for study design. A brief summary of the studies can be found in the Appendix. The majority [6] of these were excluded because they reported uncontrolled before-after study designs. Two used an interrupted time series design without enough data points pre- and post-intervention. One used a controlled before-after study design with only one control and intervention site.

### Included studies

Two controlled before-after US-based studies met our inclusion criteria. Tables 2 and 3 provide a summary of the study designs and the telemedicine applications. Lilly et al. [25] used a prospective, unblinded, stepped-wedge study over 30 months, between 2006 and 2007. The research involved one academic centre with two campuses, seven adult ICUs (medical, cardiovascular, and surgical), and 834 beds. Six thousand two hundred ninety patients were included in the study: 1529 control subjects and 4761 intervention subjects.

**Table 2:** Characteristics of included studies

Study	Country	Number of ICUs	Type of hospital	ICU model and staffing	Baseline standards	Existing IT infrastructure	Vendor	Funding source	Design (EPOC criteria)	Sample	Duration	Outcomes
Lilly et al. (2011)	USA	7 adult ICUs (medical, cardiovascular, and surgical) across 2 campuses 834 beds	Academic centre Suburban/community setting	Closed model of care Intensivist cover 24/7 Night-time cover at the discretion of the bedside ICU house staff Each ICU had a nurse manager and a medical director Bedside care provided by physicians in training, nurse practitioners, and physician assistants. 1:1 or 1:2 nurse to patient ratios Respiratory therapist also provided 24/7 cover to each unit	1 year prior to intervention: standardisation of best practice in all units (prevention of venous thrombosis, cardiovascular complications, ventilator-associated pneumonia, and stress ulcers) and introduction of ICU daily goals	1 unit: electronic record system 6 units: paper-based patient records	Philips VISICU (Baltimore, MD), APACHE® (Cerner, Kansas City, MO) with additional components provided by UMass Critical Care	University of Massachusetts	Controlled before-after study (prospective unblinded stepped-wedge design) 7 steps; duration of time intervals between steps varied from 1 day to 5 months	6290 cases: 1529 control subjects, 4761 intervention subjects	Pre-intervention: April 26, 2005–February 7, 2007 Intervention period: August 6, 2006–September 30, 2007	Primary: hospital and ICU mortality Secondary: hospital and ICU length of stay, best practice adherence, complication rates
Lilly et al. (2014)	USA	38 hospitals 56 adult ICUs (medical, surgical, coronary care, neuroscience, cardiothoracic)	Non-teaching, teaching, and teaching affiliated with a university or academic centre Rural, suburban and urban settings within 15 US states, 19 healthcare systems	Not reported	Not reported	Each ICU implemented similar technical components, including audio and video connections, an ICU-focused medical record, and software for detecting evolving physiologic instability (Koninklijke Philips N.V.)	VISICU, now owned by Koninklijke Philips N.V.	University of Massachusetts	Controlled before-after study (non-randomised, unblinded, pre-/post-design) 8-week run-in exclusion period between the start of intervention and recruitment of the first intervention subjects	118,990 cases: 11,558 control subjects, 107,432 intervention subjects	Pre-intervention: May 16, 2003–end not reported Intervention period: start not reported–December 31, 2008	Primary: hospital mortality Secondary: ICU mortality, hospital and ICU length of stay

**Table 3:** TIDieR (Template for Intervention Description and Replication) checklist [39]

Checklist	Lilly et al. (2011)	Lilly et al. (2014)
Why: Describe the rationale, theory, or goal of the elements essential to the intervention	Rationale for introduction of telemedicine linked with: 1. Earlier recognition and appropriate response to physiological deterioration (safety and timeliness) 2. Implementation of evidence-based care (effectiveness) The programme theory, i.e. how elements of the telemedicine were likely to bring about changes in outcomes not specified	Rationale for introduction of telemedicine linked with: 1. Shorter response to alarms and abnormal laboratory values 2. More rapid initiation of life-saving therapies 3. Higher rates of adherence to best practices The programme theory, i.e. how elements of the ICU telemedicine were likely to bring about changes in outcomes not specified
What: Describe the materials and procedures used in delivery of the intervention	Telemedicine technical system included: • Audio and video connectivity between bedside and remote team • Access to medical record and laboratory and radiological studies • Decision support software for detecting evolving physiologic instability, abnormal laboratory value alerts, review of response to alerts • Screening tools to help process of weaning in mechanically ventilated patients • Nurse manager rounding tool to track glycaemic control, prevention of venous thrombosis, cardiovascular complications, catheter-related bloodstream infection, ventilator-associated pneumonia, and stress ulcers • Adherence to best practice guidelines in real time Role of the off-site team • Serial review of individual patients, audits of best practice adherence, monitoring system-generated electronic alerts, and auditing bedside clinician responses to in-room alarms • Communicate with bedside clinicians or directly manage patients by recording clinician orders for tests, treatments, consultations, and management of life-support devices • Intervene when bedside clinicians’ response was delayed and patients were deemed physiologically unstable • Management of out-of-hours cases: review and assignment of case to an appropriate ICU team, patient assessment using real-time video, response to alerts and alarms, review response to the initial plan of care in real time, shared responsibility for altering the care plan if the patient’s condition fails to respond • Monitoring steps taken to remediate non-adherence and deficiencies related to inadequate documentation	Unclear how many sites already had electronic record system in place prior to the start of the programme. Telemedicine technical system provided by Koninklijke Philips N.V. (previously Philips VISICU) which included: • Audio and video connections between bedside and remote team, and electronic medical record • Decision support software for detecting evolving physiologic instability • Additional off-site team to support bedside personnel Availability of bedside documentation to the off-site team, rounding tools, and performance management varied across sites. Role of the off-site team • Serial review of individual patients, audits of best practice adherence, monitoring system-generated electronic alerts, and auditing bedside clinician responses to in-room alarms Admission, review, and intervention responsibilities varied across sites.
Who: Describe the providers of the intervention	Off-site cover Hospital staff intensivist, an ICU affiliate practitioner, a systems analyst, and one or more data clerks Integration of bedside and off-site staff • Clinical staff from the support centre also worked in the medical centre adult ICUs.	Off-site cover Intensivist available between 12 and 24 h a day, nurse available 24/7 (personal correspondence) Staffing numbers in support centre during weekdays • Intensivists 1–3 • Nurses/nurse practitioners/physician assistants 1–12 Medical director’s time dedicated to the telemedicine programme and levels of technical support provided to the programme varied across sites. ICU bedside staffing model Intensivists’ cover in ICU, medical cover out-of-hours and ICU medical director time dedicated to patient care and administration varied across sites. Integration of bedside and off-site staff Some staff from the support centre also worked at the bedside.
Where: location where the intervention took place	Off-site support centre	Not reported
When and how much: Describe the number of times the intervention was delivered and over what period of time including the number of sessions, their schedule, and their duration, intensity, or dose	• Off-site clinicians reviewed care plans for 48 % of after-hours admissions (46 % reviewed by other methods in the control period) • Total no. of alerts for physiological instability per patient per day was 6.80. Of these, 5.05 alerts were managed by bedside clinicians without telemedicine intervention and 1.75 alerts were managed with telemedicine intervention. Most interventions were initiated by the telemedicine team. • Among 24,426 interventions that affected the diagnostic or therapeutic plan, 23,943 were initiated by off-site clinicians and 483 interventions were initiated by bedside clinicians (ratio of 50:1). Among these interventions, 1633 were documented with progress notes that included a rating of the severity of the physiological disturbance; 76 % of these were classified as major (e.g. requiring initiation of a vasoactive medication).	Not reported
Tailoring or modifications: If the intervention was planned to be personalised, or was adapted during the course of the study, then describe what, why, when, and how.	Not reported	Not reported
How well: assessment of the intervention adherence or fidelity and description of any strategies used to maintain or improve fidelity	Not reported	Not reported

Prior to implementation of the intervention, all the units operated a closed model of care with 24/7 coverage with an intensivist, plus prescribing providers.^2^ The governance structure, ICU policies and procedures, call schedules, interdisciplinary rounding structure, and the numbers of provider team members were reported as constant during the study period. The critical care telemedicine system included audio and video connectivity between the bedside and the remote team, access to medical records, decision support and review of responses to alerts, screening and rounding tools, and monitoring of adherence to best practice guidelines. The team within the remotely located support centre consisted of a hospital staff intensivist, an ICU affiliate practitioner, a systems analyst, and one or more data clerks. Clinical staff from the support centre rotated through the ICUs. The clinical staff either communicated with bedside clinicians or directly managed patients by recording clinician orders for tests, treatments, consultations, and management of life-support devices. They had jurisdiction to intervene when the bedside clinicians’ response was delayed and patients were deemed physiologically unstable. They also managed the out-of-hours cases.

Lilly et al. [26] used a non-randomised, unblinded, pre-/post-assessment of telemedicine interventions over a 5-year period (2003–2008) in 56 adult ICUs. Twenty-one healthcare systems known to be implementing a telemedicine programme were invited to collect patient-level data using standardised instruments. Nineteen participating health systems enrolled patients. Dates regarding the end of baseline and start of the intervention period were not reported. Participating ICUs were geographically dispersed across 15 US states and included 38 hospitals that ranged in size from 88 to 834 beds. The ICUs served rural, suburban, and urban populations and included medical-surgical, medical, surgical, coronary care, neuroscience, and cardiothoracic units.

Data describing the characteristics of each ICU and process of care, as well as structural and organisational characteristics before and after the implementation of the telemedicine programme, were measured for each ICU using the American College of Chest Physicians ICU Telemedicine Survey instrument and reported in an adjunct paper [27]. The authors reported aggregate data from 170 ICUs in the adjunct study rather than unit-level data, so we were unable to link characteristics to the 56 units in the included study. The authors reported that each ICU implemented similar technical components, including audio and video connections, an ICU-focused medical record, and software for detecting evolving physiologic instability. Implementation changes in the process of care delivery, ICU admission procedures, rounding and governance structure, communication among caregivers, and how performance information was used, care was documented, and technical support was provided were not reported at the unit or healthcare system level.

Hospital mortality was the primary outcome for both studies with ICU mortality and ICU and hospital length of stay included as secondary outcomes. In Lilly et al. [25], case-mix and severity-adjusted hospital mortality was pre-specified as the main study outcome. They used the Simplified Acute Physiology Score (SAPS) and the Acute Physiology and Chronic Health Evaluation (APACHE III score) for severity adjustment. The authors reported unadjusted and adjusted outcomes at the unit and study levels. Their findings were analysed and compared using multivariate logistic regression. They additionally reported best practice adherence and complication rates.

Lilly et al. [26] reported pre-specified hazard ratio for dying in the hospital as the primary study outcome. They reported at the healthcare system level, adjusted and unadjusted outcomes for ICU and hospital mortality, and adjusted only data for ICU and hospital length of stay. They used the SAPS and the Acute Physiology and Chronic Health Evaluation (APACHE IV score) for acuity adjustment. Both crude and adjusted Cox proportional hazards regression models were constructed to evaluate the effects of the telemedicine interventions on hospital and ICU mortality.

### Risk of bias in included studies

Neither of the included study designs considered allocation concealment. In Lilly et al. [25], a representative sample of pre-intervention cases was obtained by identifying consecutive hospital discharge cases from an administrative database for cases managed in each of the seven ICUs. At baseline, differences in mortality and length of stay were noted within and across the ICUs. Lilly et al. [26] included patient characteristics on admission as baseline data but did not report ICU-level data, so we were unable to assess similarities in baseline outcome measurements.

In Lilly et al. [25], the intervention group had a larger percentage of medical rather than surgical patients, with slightly more abnormal laboratory and physiological values, and higher acuity scores than cases in the pre-intervention group. Similarly, the authors in the Lilly et al. study [26] noted that the telemedicine group patients had significantly higher acuity scores and predicted mortality, had a larger proportion of medical primary admission diagnoses, were less likely to have been admitted from an operating room, and had a significantly different distribution of primary admission diagnoses.

There were no reports of missing outcome measures. Missing severity scores were reported in both studies. In Lilly et al. [25], the small numbers were unlikely to bias results as the proportion of missing data was similar in the intervention and control groups. However, in Lilly et al. [26], the sizeable number of missing APACHE scores could mislead about the adjusted mortality. Blinding of primary outcomes was not undertaken in either study. Reliable outcome measures were obtained from hospital databases. All relevant outcomes listed in the ‘Methods’ section were reported. Both studies reported that electronic and manual methods of collection by abstractors yielded similar datasets and severity scores.

### Effects of intervention

Table 4 summarises the key findings from the two included studies.

**Table 4:** Effects of intervention

Mortality			
Lilly et al. (2011)	Hospital mortality	ICU mortality	
Pre-intervention (*n* = 1529)	13.6 %	10.7 %	
Telemedicine (*n* = 4761)	11.8 %	8.6 %	
Difference	1.8 %	2.1 %	
Adjusted odds ratio	OR, 0.40; CI, 0.31–0.52; *p* = .05	OR, 0.37; CI, 0.28–0.49; *p* = .003	
Lilly et al. (2014)	Hospital mortality	ICU mortality	
Control group (*n* = 11,558)	11 %	8 %	
Telemedicine (*n* = 107,432)	10 %	6 %	
Difference	1 %	2 %	
Adjusted hazard ratio	HR, 0.84; CI, 0.78–0.89; *p* = <.001	HR, 0.74; CI, 0.68–0.79; *p* = <.001	
Length of stay			
Lilly et al. (2011)	Hospital length of stay	ICU length of stay	
Pre-intervention (*n* = 1529)	13.3 days	6.4 days	
Telemedicine (*n* = 4761)	9.8 days	4.5 days	
Difference	3.5 days	1.9 days	
Adjusted hazard ratio	HR, 1.44; CI, 1.33–1.56; *p* < .001	HR, 1.26; CI, 1.17–1.36; *p* < .001	
Lilly et al. (2014)	Hospital length of stay	ICU length of stay	
Control group (*n* = 11,558)			
Telemedicine (*n* = 107,432)			
Adjusted LOS difference	15 % shorter; CI, 14–17 %; *p* < .001	20 % shorter; CI, 19–22 %; *p* < .001	
Adherence to best practice			
Lilly et al. (2011)	DVT prophylaxis	SU prophylaxis	
Pre-intervention	85 % (1299/1527)	83 % (1253/1505)	
Telemedicine	99 % (4707/4733)	96 % (4550/4760)	
Odds ratio	15.4; CI, 11.3–21.1; *p* < .001	4.57; CI, 3.91–5.77; *p* < .001	
Lilly et al. (2011)	Cardiovascular protection	VAP prevention	
Pre-intervention	80 % (311/391)	33 % (190/582)	
Telemedicine	99 % (2866/2894)	52 % (770/1492)	
Odds ratio	30.7; CI, 19.3–49.2; *p* < .001	2.20; CI, 1.79–2.70; *p* < .001	
Preventable complication rates			
Lilly et al. (2011)	VAP	CRBSI	AKI
Pre-intervention	13 % (76/584)	1.0 % (19/1529)	12 % (174/1452)
Telemedicine	1.6 % (32/1949)	0.6 % (29/4761)	12 % (540/4565)
Odds ratio	0.15; CI, 0.09–0.23; *p* < .001	0.50; CI, 0.27–0.93; *p* = .005	1.00; CI, 0.71–1.69; *p* = .38

#### Hospital and ICU mortality

The findings of Lilly et al. [25] show that after adjusting for acuity, locus of care, physiological parameters, laboratory values, and time, there was a 60 % reduction in the odds of dying in the hospital in the telemedicine group (odds ratio (OR), 0.40; 95 % CI, 0.31–0.52; *p* = .005). The ICU mortality yielded an OR of 0.37 (95 % CI, 0.28–0.49; *p* = .003) after adjustment. Subgroup analysis showed the telemedicine intervention had greater effect on hospital mortality for patients admitted after 8 pm (OR, 0.33; CI, 0.18–0.59; *p* < .001) than those admitted after 8 am (OR, 0.79; CI, 0.39–1.58).

In the Lilly et al. study [26], adjusting for relevant covariates revealed significantly lower hospital (hazard ratio (HR), 0.84; CI, 0.78–0.89; *p* < .001) and ICU (HR, 0.74; CI, 0.68–0.79; *p* < .001) HRs for patients in the critical care telemedicine group compared with the control group.

#### Hospital and ICU length of stay

Lilly et al. [25] reported that after adjustment for acuity, time trends, physiological parameters, laboratory values, and locus of care, hospital length of stay (LOS) was significantly shorter in the telemedicine group (HR for discharge, 1.44 (95 % CI, 1.33–1.56); *p* < .001). ICU LOS, after adjustment for all of the previously listed variables, yielded a HR of 1.26 (95 % CI, 1.17–1.36; *p* < .001). The reduction in LOS attributed to the telemedicine intervention was most clinically meaningful among patients who stayed in the hospital or ICU for at least 1 week. Subgroup analysis showed that cases admitted after 8 pm had longer hospital LOS (14.3 days CI 12.99–15.57 days) in the pre-intervention group than those in the telemedicine intervention group (12.4 days CI 11.22–13.58; *p* = .04) and longer ICU LOS (pre-intervention 7.7 days CI 6.77–8.63 versus post-intervention 5.5 days CI 4.86–6.08; *p* < .001).

In the Lilly et al. study [26], after adjustment, ICU LOS for the telemedicine intervention group patients was 20 % shorter (CI, 19–22 %; *p* = <.001) and hospital LOS was 15 % shorter (CI, 14–17 %; *p* = <.001) compared with control subjects. The effectiveness of the interventions for reducing LOS was clinically meaningful only among patients who remained in the hospital for at least 1 week.

#### Adherence to best practice and complication rates

In Lilly et al. [25], the telemedicine intervention period compared with the pre-intervention period was associated with higher rates of adherence to best practice guidelines for deep vein thrombosis (DVT) prophylaxis [28], stress ulcer (SU) prophylaxis [29], cardiovascular protection [30, 31], and prevention of ventilator-associated pneumonia (VAP) [32, 33]. Preventable complication rates were also lower in the telemedicine intervention period compared with the pre-intervention period for VAP and catheter-related bloodstream infection (CRBSI) but not for acute kidney injury (AKI).

#### Associations with ICU process and setting of care domains

Lilly et al. [26] reported that adjusted analyses revealed that changes in the ICU characteristics domain (OR, 0.70; CI, 0.56–0.87; *p* < .01), physician leadership domain (OR, 0.80; CI, 0.70–0.92; *p* < .01), and best practices and performance review domain (OR, 0.82; CI, 0.71–0.95; *p* < .01) were associated with significant reductions in hospital mortality. Individual components of the interventions that were reported to be associated with lower mortality, reduced LOS, or both included (1) intensivist case review within 1 h of admission, (2) timely use of performance data, (3) adherence to ICU best practices, and (4) quicker alert response times

## Discussion

Despite the increase in interest in critical care telemedicine as a potential means to improve quality and safety, the evidence base is still lacking. Our review adds to others that have found that the evidence regarding the effectiveness of telemedicine is incomplete and the quality of much of the research is poor [12, 34]. Uncontrolled before-after studies form the largest group. Most of the interrupted time series studies contain too few data collection points to account for seasonal and secular trends which might affect the data and the auto-correlation among measurements repeatedly taken over time [35]. Description of the telemedicine interventions is extremely limited making it impossible to disentangle the impact of the different elements of the telemedicine models. Very few studies consider economic outcomes, patient-reported outcomes (e.g. quality of life), or impact on work organisation and staff. There is a paucity of long-term follow-up of the effects of the intervention to allow for organisational and cultural change. We know little about patient satisfaction with critical care telemedicine and need more research on the mechanisms that contribute to changes in perceptions about safety and quality of care, particularly as telemedicine may alter relationships between patients and health professionals.

While the two included studies met the criteria for inclusion for the review, it is important to acknowledge that due to difficulties disaggregating the data between Lilly et al. [25] and Lilly et al. [26], these two studies were not independent of each other. On account of being controlled before-after studies, they were assessed as being at high risk of bias [21]. Comparison across studies and assessment of heterogeneity in terms of interventions and settings was limited by the lack of reporting detail in Lilly et al. [26].

In terms of Lilly et al.’s [25] stepped-wedge design, the variability in the length of time for the step units, which ranged from 1 day to 5 months, raises concerns about controlling for underlying temporal trends [36]. The sample size in Lilly et al. [25] was small, and the study had limited power for detecting a significant difference. Lower mortality was however associated with the telemedicine model both within ICUs over time and across ICUs during the same periods, which does suggest that the results may not be due purely to time trends [37]. Lilly et al. [25] also note that the higher acuity scores for the pre-intervention group were due in part to the staggered implementation of the telemedicine intervention and to higher rates of transfer of higher acuity medical cases from outside hospitals for the telemedicine group.

It was difficult in both studies to untangle the mechanisms involved in contributing to improved outcomes. Lilly et al. [25] attributed lower mortality to best practice adherence and decreased complication rates targeted by the intervention, although they did not report on out-of-hours versus in-hours compliance data. Telemonitoring system enables near real-time auditing and reconciliation of best practice in contrast to routine audit and feedback in critical care which is often untimely, incomplete, and not actionable [38]. Although Lilly et al. [25] suggested mortality benefits may have resulted from more rapid response to alerts for physiological instability, no baseline data was collected on triggering and response behaviour in relation to escalation. While data detailing the number of alerts and ratings of severity were generated post-intervention, this gives little indication about the timeliness or efficacy of the resulting clinical action that occurred (or did not). Neither study provided understanding about the technology’s diagnostic accuracy and the utility of the decision aids.

Lilly et al. [25] provides useful insight into bedside and off-site staffing and skill-mix and suggests team collaboration is an important variable. Lilly et al. [26] attributed earlier intensivist management, coordinated timely usage of performance information, achievement of higher rates of adherence to best practices, shorter alarm response times, more frequent interdisciplinary rounds, and a more effective ICU committee to their improved outcomes, based on the process data collected in their adjunct study [27]. They also suggest these effects are additive. However, as we did not have access to the linked data, we were unable to assess its quality.

There was a paucity of data regarding local tailoring of the programme to the local site and efforts to ensure fidelity [39]. While Lilly et al. [25] provided a detailed description of context and the programme, Lilly et al. [26] only referred to aggregate findings reported in an adjunct paper [27], so we were unable to extrapolate the ICU characteristics, structures, and processes related to the sites included in their study. The lack of standardisation of the intervention across different sites limits the ability to pool data, where sample populations, spread patterns, and definition of measures for data collection may vary. This additionally hampers the ability to compare sites for research purposes and limits the generalisability of any findings beyond their original context [40].

Organisations vary considerably in their capacity for clinical system improvement [41]. In the Lilly et al. study [25], best practices for the prevention of venous thrombosis, cardiovascular complications, ventilator-associated pneumonia, and stress ulcers were standardised and ICU daily goal setting was introduced a year before the start of the study, implying that there was opportunity for quality improvement at this stage. In the Lilly et al. study [26], all the ICUs that took part were self-selected, based on a willingness to invest in care improvement. However, the starting points of care delivery systems in terms of information technology, service improvement practices, systems, and structures [40] were not reported. The intervention period for Lilly et al. [25] was 16 months which is relatively short given the nature of organisational and cultural change associated with implementation of a complex intervention such as telemedicine.

## Conclusions

### Implications for practice

Critical care telemedicine serves a recognised need within North America, given the number of underserviced rural hospitals, lack of intensivist cover at night, and shortage of availability of intensivists. Outside the USA, providers and commissioners are showing increasing interest in telemedicine as a means of using technology effectively to re-think the way care is delivered across health economies [42]. Critical care telemedicine has important safety and quality implications as it can enable critically ill patients to stay in remote settings rather than travel to specialist centres. A networked hub and spoke structure may offer potential to level out variance in outcomes and ensure 24/7 access to specialist expertise which has implications for future workforce planning. In addition to potentially providing an additional safety net to ward-based teams, critical care telemedicine could provide a mechanism for near real-time feedback to improve situational awareness and accountability for individual actions. However, the lack of robust studies can hinder effective commissioning and can make justification of costs difficult.

### Implications for research

Our review highlights the need for further methodologically robust evaluations of critical care telemedicine. The studies that were included were not independent of each other, which highlights the importance of opening up the field to include independent evaluations of different vendors, teams, health systems, and health economies. The impact of implementation of system transformation may take some years to achieve as it necessitates cultural change [43]. Adequately powered cluster randomised trials or randomised stepped-wedge or well-designed interrupted time series studies with 12–24-month follow-up would offer a pragmatic trade-off between study design and feasibility. Researchers should consider potential modifying variables such as healthcare skill-mix ratios (both unit and off-site cover) and jurisdictional, organisational, and regulatory influences. A logic model, a diagrammatic representation of the theory of the intervention, can help document causal assumptions [44]. In order to enable understanding of telemedicine as a complex intervention, precise definitions of interventions and consideration of contextual factors and interaction effects need to be provided [39]. Detailed descriptions need to include not only the technical set-up but also how it interacts with care organisation, clinical decision-making, and professional practice to open up the black box of implementation. Future studies require accompanying process evaluations to generate theory and guide replicability and enable measurement of different end points across the causal chain and fidelity of uptake of the components of the intervention [45]. Future research also needs to incorporate an economic analysis [18]. Data needs to be collected about escalation of concerns and the timeliness or efficacy of clinical action that occurs (or does not) as a result of critical care telemedicine. Electronic surveillance systems can generate a number of false alarms with adverse consequences for patient safety [46]. Future research needs to increase understanding about the technology’s diagnostic accuracy and the utility of the decision aids. Outcome measures should be expanded to include patient- or staff-derived outcomes (e.g. quality of life, patient satisfaction, staff burnout, acceptability of telemedicine applications to providers and patients) [13]. Longer term follow-up of the impact of telemedicine on patients may help us understand its impact on patients who survive serious illness experience but experience ‘chronic critical illness’ that continues well beyond ICU discharge and often culminates in long-term morbidity and mortality [47].

### Conclusion

This review concludes that policy-makers should remain cautious about recommending increased use of, and investment in, critical care telemedicine while there is little robust evidence of clinical and economic benefit [1].

## Appendix

**Table 5:** Characteristics of excluded studies

Study	Reason for exclusion
Dhakal et al. [48]	Uncontrolled before and after design
Gupta et al. [49]	Uncontrolled before and after design, single site
Kohl et al. [50]	Controlled before and after study, only 1 intervention and control site
Morrison et al. [51]	Interrupted time series (ITS) study with less than 3 data entry points before/after each intervention
Nassar et al. [52]	Interrupted time series (ITS) study with less than 3 data entry points before/after each intervention
Sadaka et al. [53]	Uncontrolled before and after design, single site
Thomas et al. [54]	Uncontrolled before and after design
Willmitch et al. [55]	Uncontrolled before and after design
Zawade et al. [56]	Uncontrolled before and after design

## Additional file

Additional file 1: PRISMA 2009 Checklist. (DOCX 63.5 kb)

## Abbreviations

AKI: Acute kidney injury
APACHE: Acute Physiology and Chronic Health Evaluation
CI: Confidence interval
CRBSI: Catheter-related bloodstream infection
HR: Hazard ratio
ICU: Intensive care unit
OR: Odds ratio
SAPS: Simplified Acute Physiology Score
VAP: Ventilator-associated pneumonia

## Footnotes

1 Refers to a physician with intensive care certification.

2 Prescribing providers are physicians, or critical care trained nurse practitioners, or physician assistants.
